# The preventive effect of low-dose aspirin in a PPAR-γ antagonist treated mouse model of preeclampsia

**DOI:** 10.1186/s12884-022-04901-x

**Published:** 2022-07-29

**Authors:** Yongbing Guo, Yuchun Zhu, Yu Sun, Huixia Yang

**Affiliations:** Department of Obstetrics and Gynecology, Peking University First Hospital, No. 8 Sishku Street, Xicheng District, Beijing, 100034 PR China

**Keywords:** Peroxisome proliferator-activated receptor-γ, Low-dose aspirin, Preeclampsia, Dose-dependent

## Abstract

**Background:**

Preeclampsia (PE) is one of the leading causes of maternal and perinatal mortality and morbidity. Low-dose aspirin (LDA) is the most widely used drug to prevent PE, but the recommended dose of LDA varies according to different guidelines. Peroxisome proliferator-activated receptor (PPAR)-γ is involved in the formation of the placenta during pregnancy and is expressed in women with severe PE. In the present study, Our purpose was to investigate whether aspirin intervention in preeclampsia was related to PPAR-γ.

**Methods:**

We administered pregnant mice with PPAR-γ-specific antagonist(T0070907) 2 mg/kg/d at 8.5–12.5 days of pregnancy. Mice treated with T0070907 developed key features of preeclampsia. Two doses of LDA (10 mg/kg/d and 20 mg/kg/d) were administered to the mice with a PE phenotype for intervention.

**Results:**

LDA effectively decreased the increase in blood pressure in mice caused by T0070907 and decreased urinary protein levels and the urinary protein/creatinine ratio. LDA also inhibited the overexpression of endoglin and IL-β treated by T0070907. In addition, LDA evidently increased the placental weight and alleviates the degree of placental lesions of placenta and kidney. LDA alleviated the inhibition of PPAR-γ mRNA expression. The beneficial effect of 20 mg LDA was significantly better than that of 10 mg.

**Conclusions:**

(1) LDA has a preventive effect against PE treated by PPAR-γ antagonist. (2) The preventive effect of LDA against PE is dose-dependent.

## Background

PE can cause multiple organ damage and disorders in pregnant women and is one of the leading causes of maternal death worldwide [1]. The placenta is now widely considered key to the aetiology of PE. The pathophysiological manifestations of PE are trophoblast infiltration into the myometrium, poor uterine spiral artery remodelling, placental ischaemia and hypoxia induced by insufficient placental perfusion, and endothelial cell dysfunction [2]. The pathogenesis of PE is complicated, and vascular factors, metabolic regulation, oxidative stress, and inflammatory responses all play a role in this disorder [2]. Termination of pregnancy is the most effective treatment method for PE, but this can lead to iatrogenic preterm delivery, greatly increasing the morbidity and mortality of the perinatal infant. Therefore, the pathogenesis of PE and identification of effective preventive and therapeutic strategies are hot topics in the field of obstetrics.

Low-dose aspirin (LDA) is currently the preferred drug for the prevention of PE. It plays an especially important role in PE prevention in high-risk populations. In addition to the traditional cyclooxygenase (COX) pathway, non-COX-dependent pathways are involved in the preventive effect of LDA against PE [3]. In addition, the recommended dose of LDA varies according to different guidelines, ranging from 50 to 100 mg [4–7]. Further investigation of the mechanism underlying the preventive effect of LDA against PE can help determine the applicable population.

Peroxisome proliferator-activated receptors (PPARs) are ligand-activated transcription factors of which there are three subtypes: PPAR-α, PPAR-β, and PPAR-γ [8]. PPAR-γ is closely associated with normal pregnancy. PPAR-γ participates in placental formation during pregnancy and regulates vascular factors, the inflammatory response, and metabolic balance [9]. Therefore, PPAR-γ can be an important target for treating hypertension, diabetes mellitus, inflammation, and metabolic syndrome. PPAR-γ agonist activity is decreased in the peripheral blood and placenta in patients with severe PE compared with those with a normal pregnancy [10]. Fergus et al. administered T0070907 to pregnant rats at gestational days 11–15 and found that the pregnant rats exhibited typical manifestations of PE [11]. Furthermore, administration of the PPAR-γ agonist rosiglitazone to a rat model of PE induced by reduced uterine perfusion pressure (RUPP) significantly ameliorated hypertension and endothelial dysfunction [12].

In this study, pregnant mice were injected intraperitoneally with T0070907. Mice treated with T0070907 developed key features of preeclampsia. Then, two different doses of aspirin were administered, and the therapeutic effect and mechanism of action of LDA in these mice was investigated. The dose dependence of the preventive effect of LDA against PE in mice was also explored.

## Materials and methods

### Establishment of the experimental animal model and grouping

#### Experimental animals

CD-1 (ICR) mice were purchased from Beijing Vital River Laboratory Animal Technology Co., Ltd. (China), and were housed in the Experimental Animal Centre of the Peking University First Hospital. It was verified that the strains of all experimental animals conformed to international standards. The animals were housed in a clean-grade environment. All protocols used for the collection of materials from mice in this study were approved by the Ethics Committee of the Peking University First Hospital.

The CD1 mice were housed on a 12 h/12 h day/night cycle and provided access to sufficient water and food. Female mice in oestrus showed redness, wetness and thread-like changes in the vaginal orifice. Female mice in oestrus were used for mating at approximately 8 weeks. Generally, female and male mice were combined in a cage at 16:00 at a 3:1 ratio. The presence of the vaginal plug was examined in the morning of the next day, and the day on which the vaginal plug was observed was counted as embryonic day 0.5 (E0.5). Mice were intraperitoneally injected with 10% pentobarbital 10 ml/kg, and then killed by cervical dislocation at E18.5. A total of 20 sexually mature male mice and 76 female mice were used in this study. Three female mice died during model establishment.

#### Model establishment and experimental animal grouping

Pregnant mice were injected intraperitoneally with T0070907 2 mg/kg/d at 8.5–12.5 days of pregnancy and the mice developed a PE phenotype.. Next, different doses of LDA (10–20 mg/kg/d) were administered to the T0070907 treated mice.

Mice with vaginal plugs (E0.5) were randomly divided into four groups and seven subgroups:

1. The control group (*N* = 16), which was divided into two subgroups, i.e., a) the intraperitoneal group (*N* = 8) and b) the intragastric group (*N* = 8);
2. The PPAR-γ antagonist group (antagonist group, *N* = 8);
3. The PPAR-γ antagonist + LDA group (*N* = 16), which was divided into two subgroups, ie., a) the PPAR-γ antagonist + 10 mg/kg/d LDA (antagonist + LDA 10 mg) group (*N* = 8) and b) the PPAR-γ antagonist + 20 mg/kg/d LDA (antagonist + LDA 20 mg) group (*N* = 8);
4. The LDA group (*N* = 16), which was divided into two subgroups, i.e., a) LDA 10 mg group (*N* = 8) and b) LDA 20 mg group (*N* = 8).

Pregnant mice in the PPAR-γ antagonist group were given a T0070907 (2 mg/kg) by intraperitoneal injection every day from E8.5-E12.5. Mice in the antagonist + LDA group were given different doses of LDA every day starting at E0.5 by gastric gavage and a PPAR-γ antagonist (2 mg/kg) every day from E8.5 to E12.5 by intraperitoneal injection. Pregnant mice in the control group were treated with normal saline at the same volume as LDA or the PPAR-γ antagonist. Mice in the LDA group were given different doses of LDA every day starting at E0.5 by gastric gavage.

#### In vivo administration of the PPAR-γ antagonist

A PPAR-γ specific antagonist (T0070907, Abcam) was given at a dose of 2 mg/kg every day from E8.5-E12.5 by intraperitoneal injection. Dosing was performed according to the initial weight of the animals. The timing and dose of T0070907 used in this study was chosen on the basis of a previously published study [11, 13, 14]. The dose of T0070907 for the mice was calculated according to the FDA conversion table [15].

### Sample collection

#### Measurement of the systolic blood pressure (SBP) and the diastolic blood pressure(DBP)of pregnant mice

Blood pressure was measured using a noninvasive mouse tail sphygmomanometer (Softron BP-2010A). Blood pressure was measured at 8:00–11:00 in the morning at E0.5 and E17.5.

#### Analysis of 24-h urine from mice

Mice were placed individually in metabolic cages at E17.5. The mice were given normal drinking water and provided powdered mouse chow. The metabolic cages were completely sealed, and vapourization of mouse urine was minimized as much as possible during testing to determine the normal urinary output of the mice over 24 h. The urine retention devices at the bottom of the metabolic cages were removed after 24 h. The urine volume was measured, and the urine was aliquoted and stored.

#### Collection of mouse blood

After collection of mouse urine at E18.5, the mice were sacrificed by cervical dislocation, and blood samples were collected immediately through the abdominal aorta. The mice were placed on a surgical frame in the supine position. A ventral midline incision was made layer by layer in the abdominal cavity to fully expose the abdominal aorta. Blood was aspirated with a sterile syringe and transferred to a blood collection tube with an EDTA to prevent haemolysis. The blood collection tube was centrifuged at 3000 rpm for 10 min to collect the supernatant.

### Polymerase chain reaction (PCR)

PPAR-γ mRNA levels in the placentas of pregnant mice were measured by PCR. Total RNA was extracted from the placental tissues of pregnant mice and reverse-transcribed into cDNA. The primer sequences were as follows: PPAR-γ: upstream, 5’-AGACCACTCGCATTCCTTT-3’, and downstream, 5’-CACAGACTCGGCACTCAAT-3’; GAPDH: upstream, 5’-TGGTGAAGGTCGGTGTGAAC-3’, and downstream, 5’-GCTCCTGGAAGATGGTGATGG-3’. The PCR conditions were as follows: 95 °C for 30 s for predenaturation and 40 cycles of denaturation at 95 °C for 5 s and annealing at 60 °C for 30 s. F = 2^−∆∆Ct^ was used to calculate the relative mRNA level of the target gene.

### Enzyme-linked immunosorbent assay (ELISA)

Interleukin (IL)-1β, endoglin, and creatinine levels in the peripheral blood and the creatinine level in the urine of pregnant mice was measured by ELISA. The concentrations of endoglin and IL-1β in plasma samples from mice were measured using reagent kits from Elabscience. The urine creatinine level in mice was measured using a reagent kit (DICT-500: BioAssay Systems, CA, USA).

### Pathological staining

Mouse kidney and placental tissues were fixed, dehydrated, cleared, embedded, and sectioned into 2-μm sections. The sections were stained with haematoxylin and eosin (HE) and periodic acid–Schiff, methenamine silver, and Masson’s trichrome.

In this study, each group randomly selected three mice for pathological staining and one kidney and three placentas were chosen completely at random in each mouse. Nine placental pathological slides of each group were examined, which was made by full—layer slitting. The pathological staining analysis by reviewers blinded to the treatment groups. We used the Image Pro-Plus software to compare the size of the lesion area in the screenshots of the field with the same magnification. And the degree of renal pathology was indicated by ( ±).

### Statistical methods

All data are expressed as the median (minimum, maximum). Statistical analyses were performed using the Mann–Whitney test and the Kruskal–Wallis test. *P* < 0.05 was considered significant. Statistical graphs were created using GraphPad Prism 5.0 software.

## Results

### Blood pressure

**Fig. 1:**
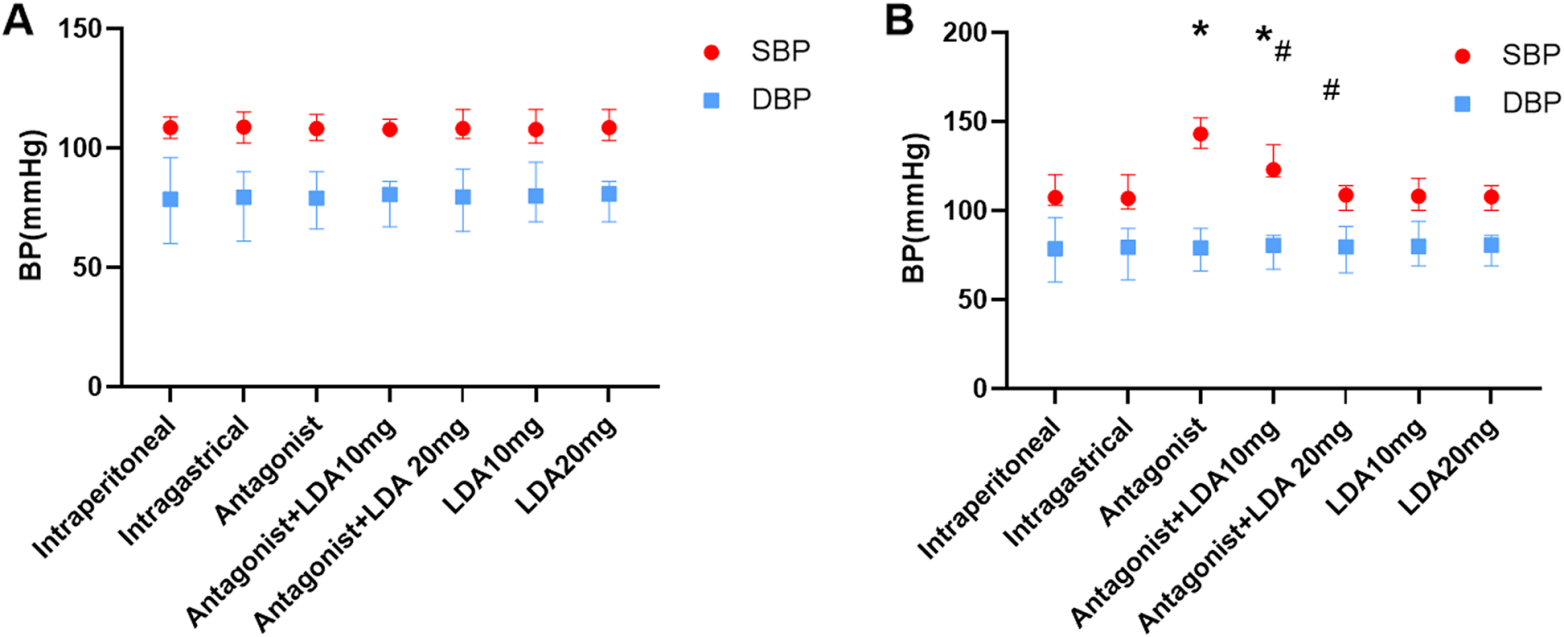
Blood pressure of E0.5 and E17.5 in each group

The mean systolic and diastolic blood pressure at E17.5 was significantly higher in the PPAR-γ antagonist-treated group than in the control group (Fig. 1). SBP was 24.5% lower in the antagonist + LDA 20 mg group and 13.9% lower in the antagonist + LDA 10 mg group than in the antagonist group. In addition, compared with the antagonist group, the DBP of the antagonist + LDA 20 mg group was reduced by 7.1%, and that of the antagonist + LDA 10 mg group was reduced by 4.7%. It is indicated that both 10 mg/kg/d LDA and 20 mg/kg/d LDA lowered blood pressure in mice with PE. After treatment with 20 mg/kg/d LDA, the MABP of mice with PE returned mostly to normal at E17.5 (Table 1).

**Table 1:** Blood pressure of E0.5 and E17.5 in each group

	Intraperitoneal	Intragastrical	Antagonist	Antagonist + LDA10mg	Antagonist + LDA 20 mg	LDA10mg	LDA20mg
E0.5 SBP (mmHg)	108.5 [104,113]	108.5 [102,115]	108.5 [103,114]	107 [105,112]	107.5 [104,116]	106.5 [102,116]	107 [103,116]
E0.5 DBP (mmHg)	78.5 [60,96]	79.4 [61,90]	79 [66,90]	80.5 [65,91]	79.5 [69,94]	106.5 [102,116]	107 [103,116]
E17.5 SBP (mmHg) E17.5 DBP (mmHg)	105 [103,120]	106 [101,120]	142 [135,152]	122 [119,137]	112 [100,114]	109 [100,118]	109 [100,114]
	75.3 [60,89]	75.8 [61,86]	85.6 [81,90]	81.8 [77,86]	79.5 [69,85]	76.6 [103,116]	75.8 [66,86]

Each group contained 8 mice. A E0.5 SBP and DBP level of mice in different treatment groups.B E17.5 SBP and DBP of mice in different treatment groups.The result is expressed as the median, the largest value and minimum value. Mann–whitney test and Kruskal–Wallis test were used for statistical analysis.*, compared with control group *P* < 0.05. #, compared with Antagonist group *P* < 0.05.

### Level of 24-h urine protein and the urine protein/creatinine ratio

**Fig. 2:**
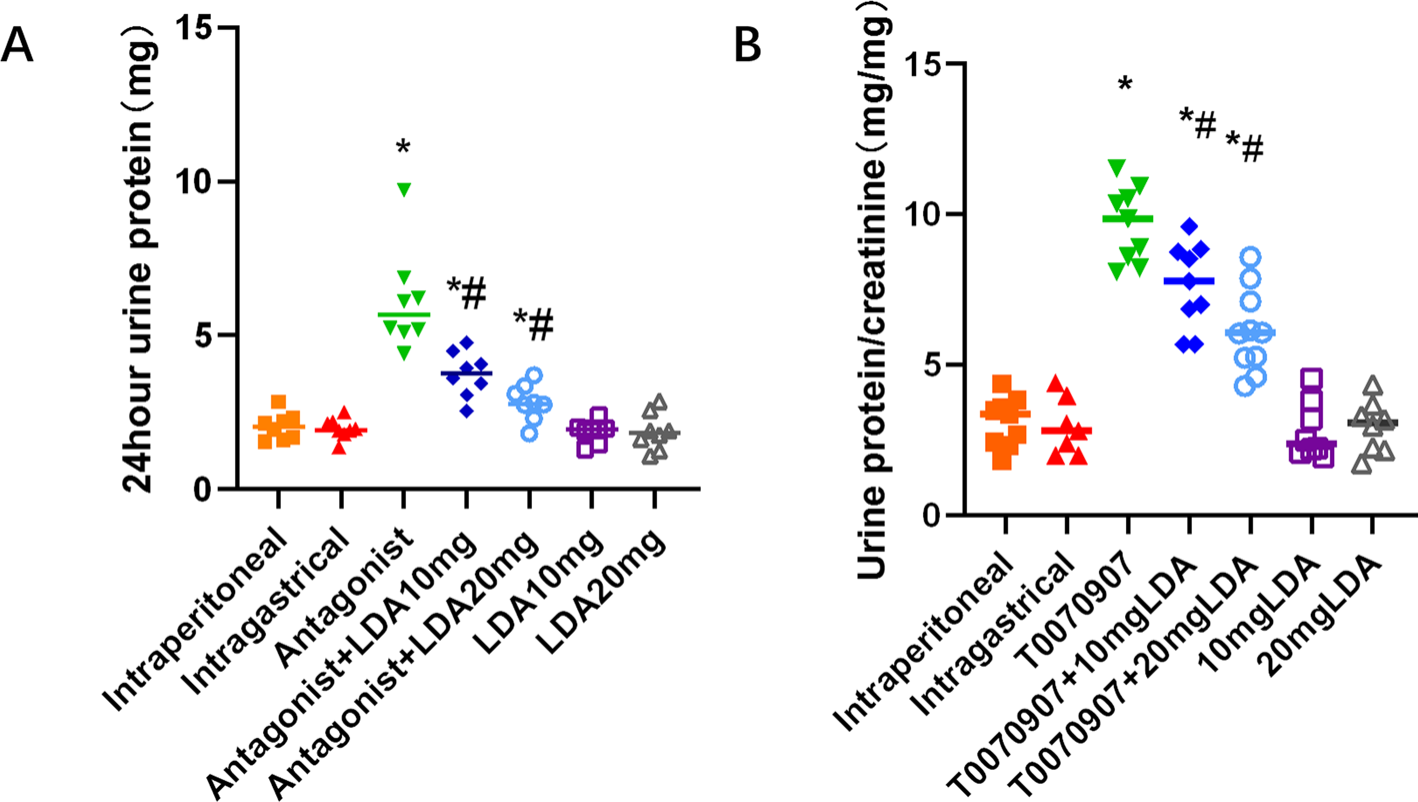
24-h Urine protein and urine protein creatinine ratio of E18.5 in each group

The total 24-h urine protein level and the urine protein/creatinine ratio were significantly higher in the T0070907-treated group than in the control group (Fig. 2). The total 24-h urine protein level was decreased by 51% in the antagonist + LDA 20 mg group and 33% in the antagonist + LDA 10 mg/kg group compared with the antagonist group. The urine protein/creatinine ratio of the antagonist + LDA 20 mg group was decreased by 40%. These differences were all significant. The urine protein/creatinine ratio of mice in the antagonist + LDA 10 mg group was 13% lower than that of mice in the antagonist group, but this difference was not significant (Table 2).

**Table 2:** 24-h Urine protein and urine protein creatinine ratio of E18.5 in each group

	Intraperitoneal	Intragastrical	Antagonist	Antagonist + LDA10mg	Antagonist + LDA 20 mg	LDA10mg	LDA20mg
24 h Urine protein(mg)	2.05 [1.54,2.84]	1.94 [1.37,2.51]	5.68 [4.41, 9.72]	3.78 [2.54,4.77]	2.78 [1.82,3.71]	1.95 [1.30,2.39]	1.83 [1.10,2.87]
Urine protein/creatinine ratio	3.43 [1.80,4.37]	2.59 [1.98,4.39]	9.37 [8.09,11.53]	8.15 [5.68,9.59]	5.64 [4.29,8.58]	2.35 [1.93,4.54]	3.08 [1.73,4.34]

Each group contained 8 mice. A 24-h Urine protein results of E18.5 in different treatment groups. B Urine protein/creatinine ratio of E18.5 in different treatment groups. The result is expressed as median, maximum, and minimum. Mann–whitney test and Kruskal–Wallis test were used for statistical analysis. *, compared with the control group *P* < 0.05. #, compared with Antagonist group *P* < 0.05.

### Endoglin expression

**Fig. 3:**
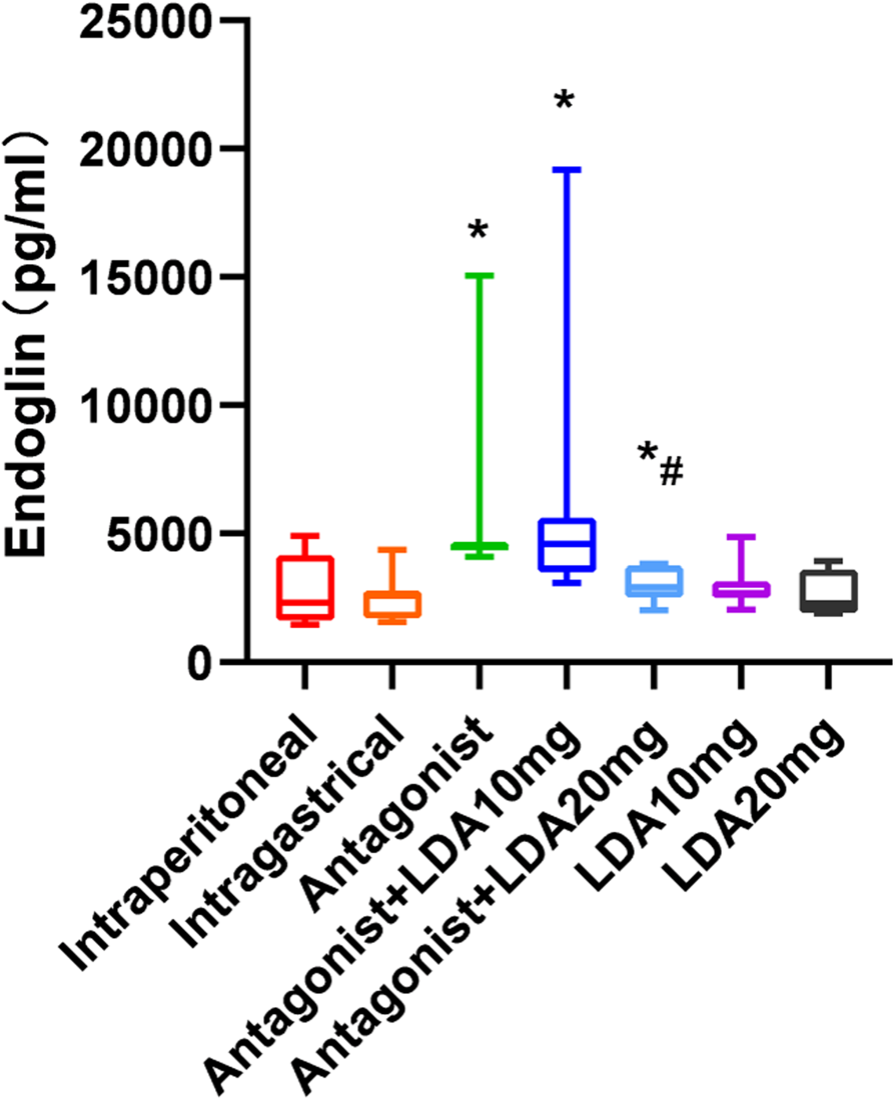
Endoglin concentration in peripheral blood of mice in each group

The expression of peripheral endoglin was significantly higher in the T0070907-treated group than in the control group (Fig. 3). The endoglin concentration in the peripheral blood of mice in the antagonist + LDA 10 mg group was slightly higher than that the peripheral blood of mice in the antagonist group, but this difference was not statistically significant. The endoglin concentration in the peripheral blood of mice in the antagonist + LDA 20 mg group was significantly lower than that in the peripheral blood of mice in the antagonist group (Table 3).

**Table 3:** Endoglin and IL-1β concentration in peripheral blood of mice in each group

	Intraperitoneal	Intragastrical	Antagonist	Antagonist + LDA10mg	Antagonist + LDA 20 mg	LDA10mg	LDA20mg
Endoglin(pg/ml)	2288.64[1458.49,4910.49]	2538.89[1579.05,4358.41]	4467.68[4096.22,15,044.72]	4866.85[3090.87,19,170.41]	2904.26[2022.26,3827.50]	2616.03[2058.18,4855.63]	2909.29[1913.42,3935.34]
IL-1β (pg/ml)	57.07 [27.82,81.12]	62.42 [41.33,123.90]	100.32 [36.97,137.36]	46.27 [19.73,341.09]	34.88 [13.52,64.99]	19.14 [7.18,104.55]	35.61 [3.18,91.26]

Each group contained 8 mice. The results are expressed as median, maximum, and minimum.Mann–whitney test and Kruskal–Wallis test were used for statistical analysis.*, compared with control group *P* < 0.05.#, compared with Antagonist group *P* < 0.05.

### IL-1β concentration in peripheral blood

**Fig. 4:**
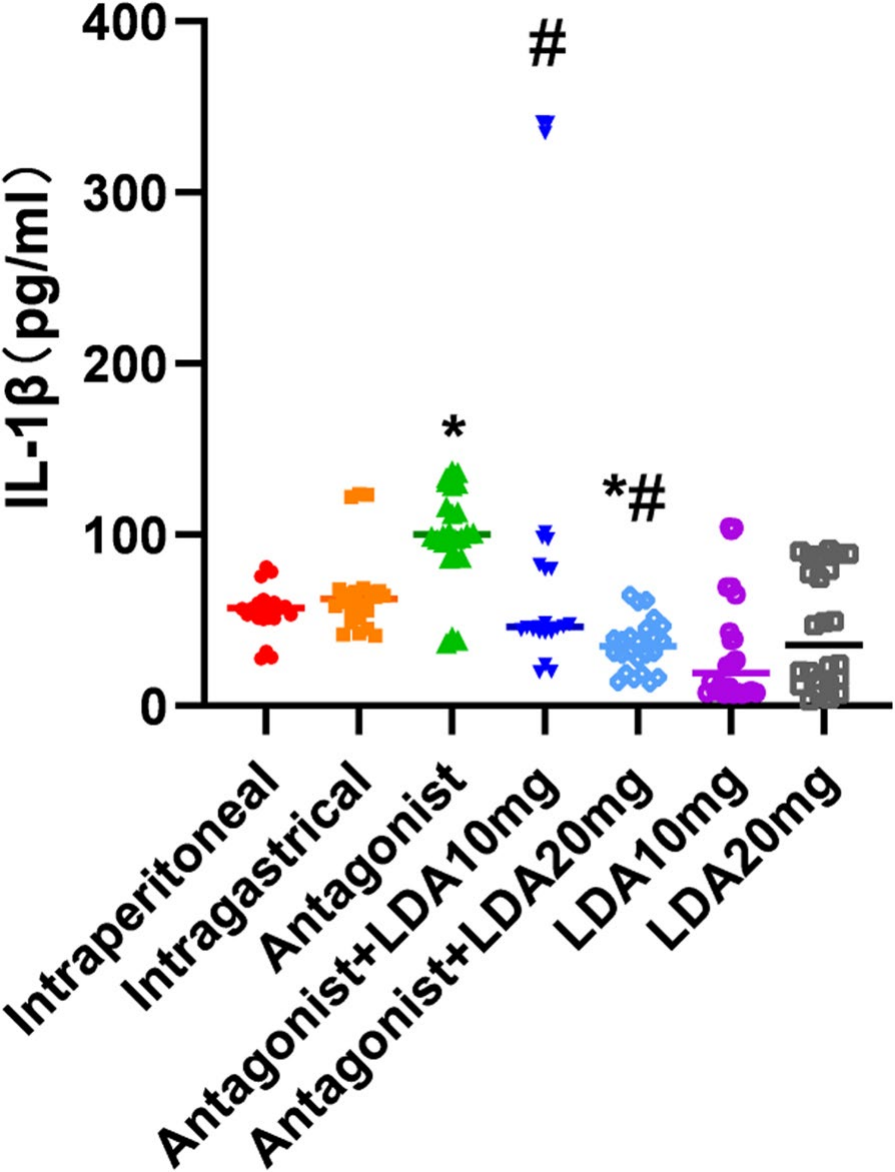
IL-1 concentration in peripheral blood of mice in each group

IL-1β expression in the peripheral blood of mice was significantly higher in the T0070907-treated group than in the control group (Fig. 4). The IL-1β level in the peripheral blood of mice in the antagonist + LDA 10 mg group was decreased compared with that in the antagonist group and was decreased even further in the antagonist + LDA 20 mg group. The IL-1β level in peripheral blood was similar between the control group and the antagonist + LDA 10 mg group. The IL-1β level in the peripheral blood of mice in the antagonist + LDA 20 mg group was lower than that in the peripheral blood of mice in the control group (Table 3).

Each group contained 8 mice. The results are expressed as median, maximum, and minimum.Mann–whitney test and Kruskal–Wallis test were used for statistical analysis.*, compared with control group *P* <  0.05. #, compared with Antagonist group *P* < 0.05.

### PPAR-γ mRNA expression in the placenta

**Fig. 5:**
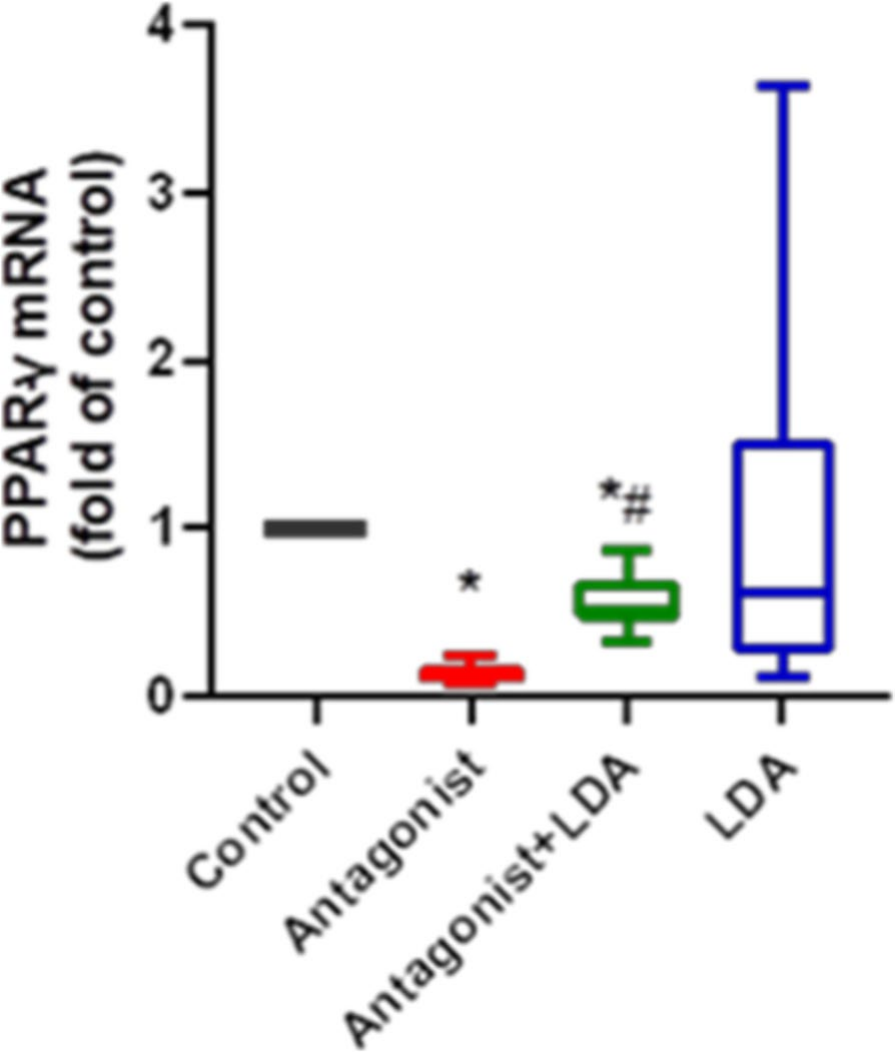
Expression of PPAR- mRNA in peripheral blood of mice in each group

PPAR-γ mRNA expression in mice in the antagonist group was significantly reduced compared with that in mice in the control group (Fig. 5). The decrease in PPAR-γ mRNA expression in the placentas of mice was significantly ameliorated in the antagonist + LDA group(10 mg + 20 mg) compared with the antagonist group.

The results are expressed as median, maximum, and minimum.Mann–whitney test and Kruskal–Wallis test were used for statistical analysis.*, compared with control group *P* < 0.05.#, compared with Antagonist group *P* < 0.05. Antagonist group each has 8 mice. Control group,Antagonist + LDA group and LDA group each has 16 mice.

### Placental weight

**Fig. 6:**
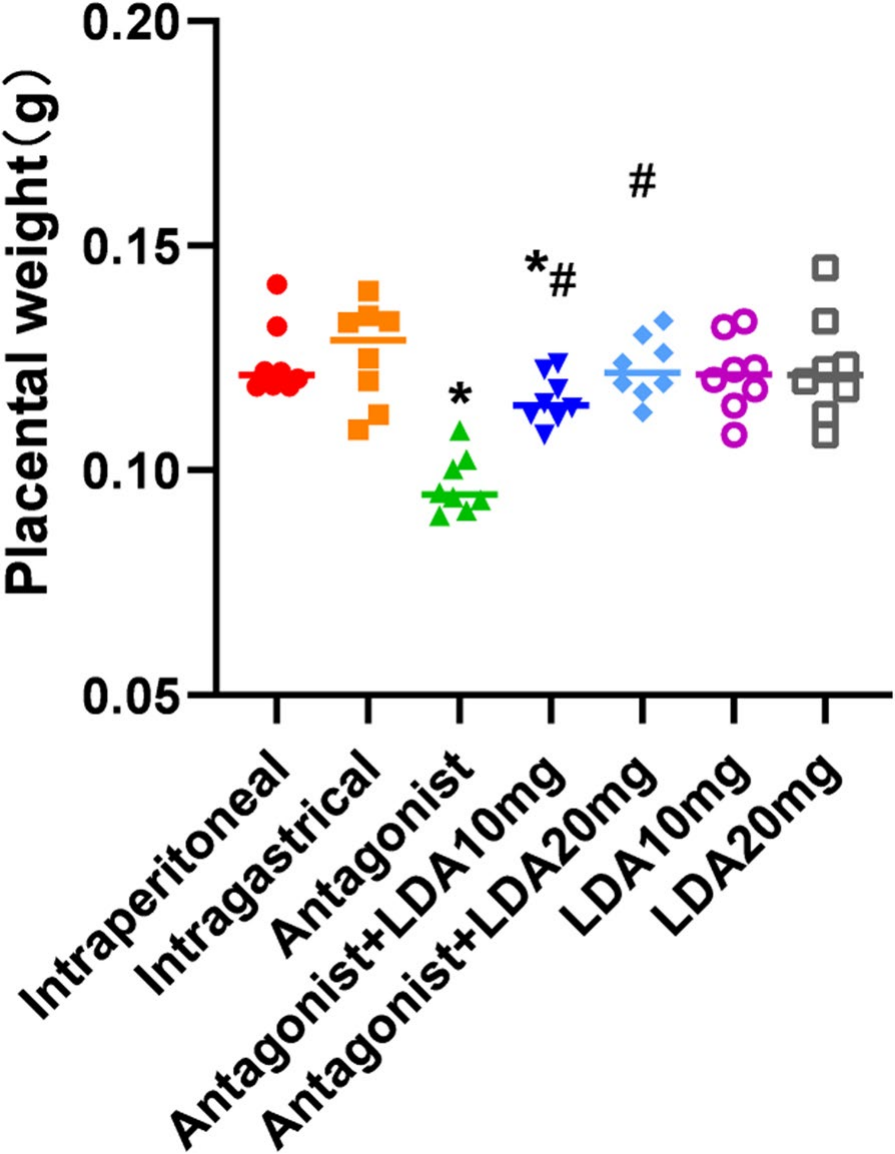
Placental weight of mice in each group

The placental weight of mice in the antagonist group was 23% of that of mice in the control group (Fig. 6). The placental weight of mice in the antagonist + LDA 10 mg group was significantly increased compared with that of mice in the antagonist group and was increased even further in the antagonist + LDA 20 mg group. Placental weight was similar between the control group and the antagonist + LDA 20 mg group but was significantly different between the control group and the antagonist + LDA 10 mg group. These results show that aspirin could help ameliorate the reduction in placental weight in mice with PE induced by a PPAR-γ antagonist in a dose-dependent manner. The preventive effect of daily administration of 20 mg/kg/d LDA was much better than that of daily administration of 10 mg/kg/d LDA (Table 4).

**Table 4:** Placental weight of mice in each group

	Intraperitoneal	Intragastrical	Antagonist	Antagonist + LDA10mg	Antagonist + LDA 20 mg	LDA10mg	LDA20mg
Placental weight(g)	0.1213[0.1187,0.1414]	0.1290[0.1091,0.1400]	0.0946[0.0900,0.1090]	0.1145[0.1080,0.1238]	0.1217[0.1129,0.1333]	0.1214[0.1079,0.1332]	0.1212[0.1078,0.1451]

Each group contained 8 mice. The results are expressed as median, maximum, and minimum.Mann–whitney test and Kruskal–Wallis test were used for statistical analysis.*, compared with control group *P* < 0.05. #, compared with Antagonist group *P* < 0.05.

### Pathological analysis of the mouse placenta

**Fig. 7:**
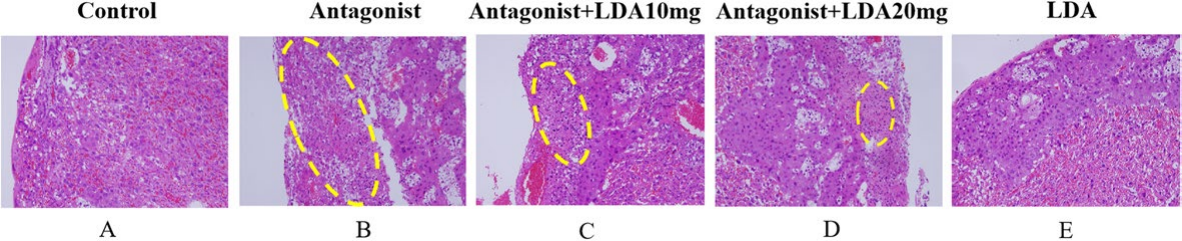
Pathological results of placenta of mice in each group (HE, × 200)

The area of placental infarction was significantly decreased and the degree of neutrophil infiltration reduced in mice in the antagonist + LDA 10 mg group compared to mice in the antagonist group (Fig. 7 and Table 5). The placental structure of mice in the antagonist + LDA 20 mg group was mostly normal, and only a few small areas of tissue infarction could be observed on the edge of the foetal membrane. There was significantly less infarcted placental tissue and a significantly smaller infarct range and inflammatory exudate range in the antagonist + LDA 20 mg group than in the antagonist group. Placental pathology was similar between the LDA group and the control group.

**Table 5:** Proportion of placenta infarcted lesions in each group

	Intraperitoneal	Intragastrical	Antagonist	Antagonist + LDA10mg	Antagonist + LDA 20 mg	LDA10mg	LDA20mg
Proportion of placenta infarcted lesions	0%	0%	61%	39%	23%	3%	5%

Figure A-E is divided into control group, Antagonist group, Antagonist + LDA10mg group, Antagonist + LDA20mg group and LDA.Pathological results of placenta of mice in group A.HE staining was used in all cases, and the magnification was 200 times.The dotted yellow line indicates inflammatory exudation with infarction parts. 3 mice were randomly selected from different groups, with 3 placentas in each mouse and 3 random fields in each placenta for pathological staining.

### Pathological analysis of the mouse kidney

**Fig. 8:**
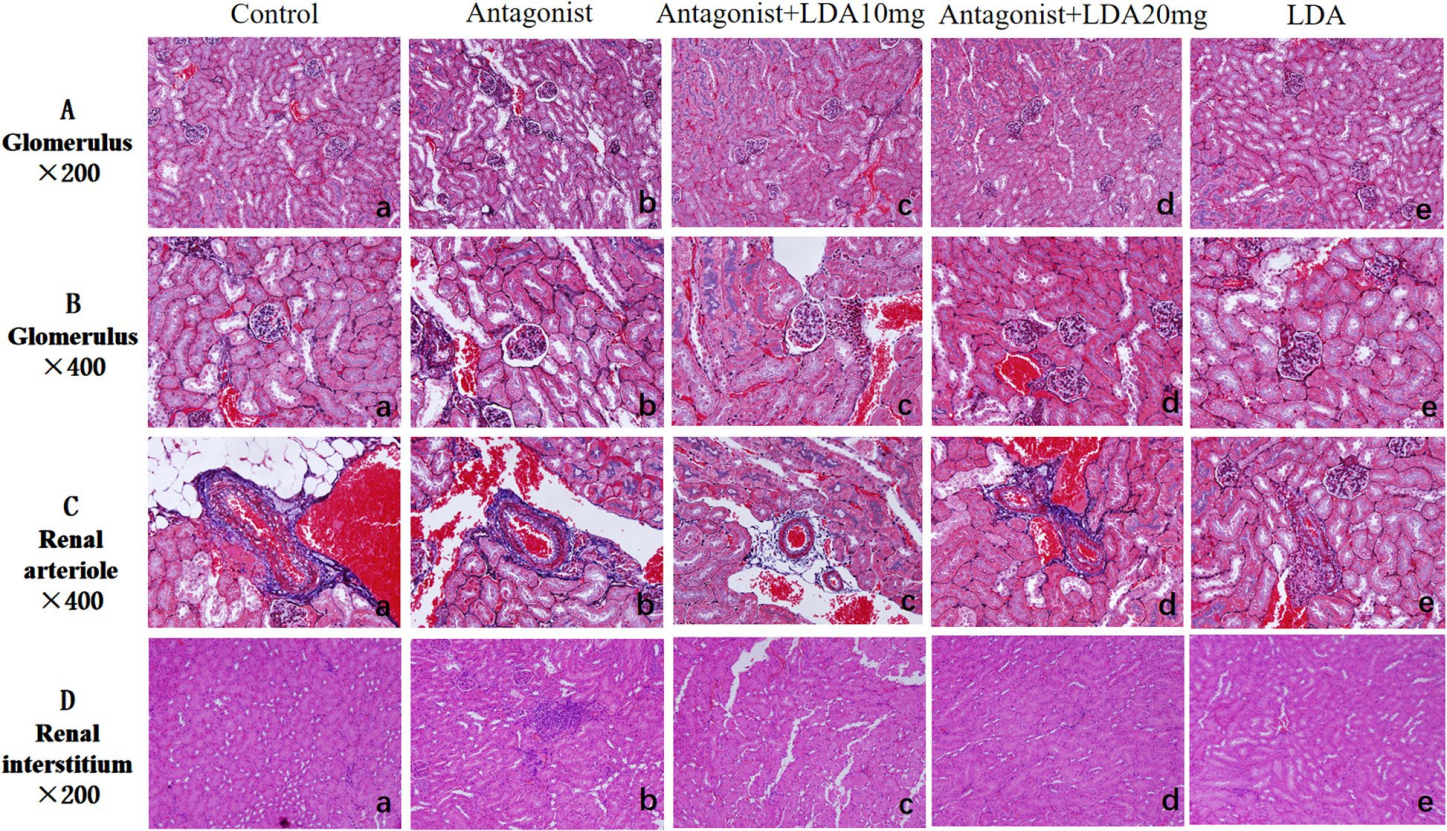
Renal pathological results of mice in each group

Administration of 10 mg/kg/d LDA following antagonist treatment ameliorated glomerular basement membrane shrinkage, decreased the dilation of Bowman's capsule, and decreased renal interstitial neutrophil infiltration (Fig. 8 and Table 6). In the antagonist + LDA 20 mg group, the glomerular basement membrane did not exhibit significant shrinkage, Bowman’s capsule dilation was not significant, and there was only local renal interstitial neutrophil infiltration. Glomerular and renal interstitial pathology was similar between the LDA group and the control group. Pathological analysis of the renal arterioles did not reveal significant differences between the five groups of mice.

**Table 6:** The degree of change in the glomeruli of each group

	Intraperitoneal	Intragastrical	Antagonist	Antagonist + LDA10mg	Antagonist + LDA 20 mg	LDA10mg	LDA20mg
The degree of change in the glomeruli	-	-	+ + +	+ +	+	-	-

A(A-E) is divided into control group, Antagonist group, Antagonist + LDA10mg group, Antagonist + LDA20mg group, and LDA.The pathological results of glomerular hexamethylamine silver sheath staining with Masson staining were all 200 times magnified.B (A-E) showed small kidneys in the above five groups.The histopathological results of bulb hexylamine silver sheath staining with Masson staining were all 400 times magnified.C (A-E) was silver hexamethylamine staining of renal arterioles in five groups of mice.Pathologic results of Masson staining showed that the magnification was 400 times.D (A-E) was the results of HE staining in the renal interstitium of the five groups of mice, with all amplification multiples.For 200 times. 3 mice were randomly selected from different groups, with 1 kidney in each mouse and 3 random fields in each placenta for pathological staining.

## Discussion

### The relationship between PPAR-γ and PE

PPAR-γ plays an important role in pregnancy. In this study, mice treated with T0070907 developed key features of preeclampsia, including elevated mean arterial blood pressure, proteinuria, endothelial dysfunction. This is consistent with previous reports [11–16].In addition, we found that the placental weight decreased and the pro-inflammatory factors increased in the antagonist group. The results indicated that PPAR-γ participates in the pathogenesis of PE.

Previous literature reports examination of the preeclamptic placenta reveals numerous placental infarcts and arterial sclerosis. Infarction may be due to maternal vascular malperfusion [17, 18]. Placental hypoperfusion may lead to placental ischemia and contribute to the development of preeclampsia. In this study, we found a large number of infarcts in placental sections of mice treated with T0070907, which consistent with the symptoms of preeclampsia. The results indicated that PPAR-γ production was suppressed, leading to a reduction in the placental weight and necrotizing injury. Kubota et al. also showed that PPAR-γ deficiency resulted in abnormal placental development [19].

In this study, the level of the proinflammatory cytokine IL-1β in the peripheral blood was significantly higher in the PPAR-γ antagonist group than in the other groups. This result indicated that suppression of PPAR-γ production resulted in higher levels of proinflammatory cytokines in the peripheral blood. At this lower level, PPAR-γ could not normally regulate inflammatory responses, eventually causing PE manifestations in the mice.

### Significant amelioration of PPAR-γ antagonist-induced PE phenotypes by LDA

This study showed that LDA intervention significantly ameliorated hypertension treated by T0070907 in pregnant mice. In addition, the 24-h urine protein level and urine protein/creatinine ratio were significantly reduced in the LDA-treated groups than in the T0070907-treated group. Furthermore, LDA effectively decreased the production of proinflammatory factors and antiangiogenic factors, reducing the levels of these factors to levels observed in normal pregnancy. Pathological analysis of placental and kidney tissues from mice in the different treatment groups indicated that LDA significantly alleviated kidney and placenta infarcts treated by T0070907.

### The mechanism of action of aspirin in the prevention of PE treated by T0070907 in mice

PPAR-γ mRNA expression in the placentas of mice treated by T0070907 was downregulated, which is consistent with previous research by McCarthy et al. [11]. In our study, preventive administration of LDA significantly relieved the downregulation of PPAR-γ mRNA. In addition, LDA was significantly ameliorated the overexpression of antiangiogenic factors and inflammatory cytokines treated by T0070907 and pathological injury to the placenta and kidney. In summary, LDA significantly ameliorates the PE promoted by T0070907 through various pathways. Therefore, this study suggests that LDA prevents PE through the regulation of PPAR-γ expression.

A study by Lehmann et al. showed that nonsteroidal anti-inflammatory drugs (NSAIDs) can activate PPAR-γ [20]. That study suggests that at certain concentrations, NSAIDs can inhibit or induce adipogenesis by activating the PPAR-γ pathway. So combined with this experiment, we propose the possibility that aspirin, as an NSAID, might exert biological effects at certain concentrations by activating the PPAR-γ pathway. However, further research is still needed.

PPAR-γ is a nuclear hormone receptor superfamily member that has many functions in the body. At the cellular level, PPAR-γ agonists have anti-inflammatory, anti-proliferative, anti-fibrotic, and anti-apoptotic functions. At the system level, PPAR-γ agonists can induce haemodynamic changes in humans and animals to produce anti-hypertensive effects [9]. PPAR-γ might have the potential to relieve hypertensive disorders in pregnancy [21]. McCarthy et al. found that application of the PPAR-γ agonist rosiglitazone to the reduced uterine perfusion pressure(RUPP) rats alleviated the PE-like pathological symptoms to a certain degree [12]. Thus, this study proposes that PPAR-γ agonists might help relieve PE. In addition, McCarthy et al. found that PPAR-γ agonists improved pregnancy outcomes of mice with PE, mainly through the activation of haem oxygenase-1 (HO-1), which is consistent with a study by Cudmore et al. showing that PPAR-γ agonists reduced the production of ROS and sFlt-1 by upregulating HO-1 expression to reduce oxidative stress in the bodies of PE patients and restore the balance of angiogenic factors [11, 22]. Other studies have confirmed that activation of PPAR-γ can restore nitric oxide (NO) bioavailability [23]. Martens et al. showed that activation of PPAR-γ promoted NO production in human aortic endothelial cells [24].

PPAR-γ agonists can not only interfere with oxidative stress and angiogenesis pathways but also effectively reduce the intensity of inflammation in the bodies of PE patients. Hofmann et al. showed that PPAR-γ activation in monocytes and macrophages reduced the production of cytokines such as tumour necrosis factor-α, IL-1β, and IL-6 [25]. PPAR-γ activation can also inhibit the proinflammatory effect of platelets and suppress platelet aggregation and thromboxane A2 (TXA2) release [26]. Therefore, PPAR-γ agonists could help to restore the balance of TXA2/PGI2 in PE patients.

It has been confirmed that PPAR-γ agonists have protective effects on the kidney and can effectively reduce proteinuria. Sugawara showed that PPAR-γ agonists could reduce blood pressure, protect vascular endothelial functions, cause vasodilation of the glomerular arterioles, and reduce proteinuria [27]. PPAR-γ agonists were shown to protect podocytes in a rodent model of kidney disease and glomerular hypertension [28]. Especially after podocyte injury, PPAR-γ activation plays a key protective role. Glomerular podocytes are key cells for the prevention of proteinuria, kidney failure, and cardiovascular disease.

In this study, LDA was administered to mice with PE treated by T0070907 and was found to significantly ameliorate hypertension and proteinuria. In addition, LDA effectively rescued the downregulation of PPAR-γ mRNA expression. Therefore, we believe that LDA could prevent PE by regulating PPAR-γ expression. PPAR-γ mRNA expression in the mouse placenta varied widely in the group that received preventive administration of LDA. This reflects the large individual differences that could confound the preventive use of LDA in clinical practice; therefore, more accurate confirmation of the target population for LDA treatment is clinically significant. Thus, whether LDA prevents PE through the activation of the PPAR-γ pathway and its specific action mechanism require further investigation.

### Dose effect of aspirin

The results indicated that 20 mg/kg/d LDA has significant preventive effects against PE treated by T0070907. These results suggest that the preventive effect of aspirin on PE in mice was dose-dependent.

Precious observational study has also shown that daily administration of 100–160 mg LDA might be necessary to prevent PE and that 60–80 mg LDA might not be enough to prevent PE [29]. The above views are all consistent with the conclusions of this study.

## Conclusion

In this study, mice treated with T0070907 developed key features of preeclampsia. In mice PPAR-γ participates in the pathogenesis of PE in three ways: by regulating the formation of the placenta, inflammatory factors, and vascular factors. LDA has preventive effects in mice with PE treated by the PPAR-γ antagonist. LDA might exert its PE-preventing effects through the regulation of PPAR-γ production. The effect of LDA against PE is dose-dependent.

## Data Availability

The datasets obtained and/or analyzed during the current study are available. From the corresponding author on reasonable request.

## Abbreviations

LDA: Low-dose aspirin
IL: Interleukin
PE: Preeclampsia
COX: Cyclooxygenase
PPARs: Peroxisome proliferator-activated receptors
PCR: Polymerase chain reaction
ELISA: Enzyme-linked immunosorbent assay
HE: Haematoxylin and eosin
NSAIDs: Non-steroidal anti-inflammatory drugs
PGs: Prostaglandins
RUPP: Reduced uterine perfusion pressure
HO-1: Haeme oxygenase-1
NO: Nitric oxide
TXA2: Thromboxane A2
